# Recent advances in understanding and preventing peanut and tree nut hypersensitivity

**DOI:** 10.12688/f1000research.14450.1

**Published:** 2018-10-30

**Authors:** Ayan Kusari, Allison Han, Lawrence Eichenfield

**Affiliations:** 1 Departments of Pediatric and Adolescent Dermatology, Rady Children’s Hospital, San Diego, California, USA; 2 Department of Dermatology, University of California, San Diego School of Medicine, San Diego, California, USA

**Keywords:** Peanut Allergy, Tree Nut Allergy, Nut Hypersensitivity, Oral Tolerance, LEAP study, NIAID guidelines

## Abstract

Peanut allergy, the most persistent and deadly of the food allergies, has become more prevalent worldwide in recent decades. Numerous explanations have been offered for the rise in peanut allergy, which has been more pronounced in Western, industrialized nations. In infants who are at increased risk of peanut allergy, new evidence indicates that early introduction of peanuts can help prevent allergy development. This counterintuitive finding directly contradicts the previously established practice of peanut avoidance for high-risk infants but is supported by clinical and basic science evidence. Here, we review the literature contributing to our evolving understanding of nut allergy, emphasizing the translation of this work to clinical practice.

## Introduction

The global impact of food allergy has increased in the past few decades, and industrialized countries appear to be disproportionately affected. Food allergy prevalence is marked in the US, where incidence is as high as 10% in 1-year-olds ^1^ and estimates of economic impact are as high as $25 billion per year ^2^. Anaphylaxis, which is caused by IgE-mediated degranulation of mast cells and basophils, is the most serious form of food hypersensitivity and may result in hypotension, shock, and bronchospasm. Admissions for food anaphylaxis in children have doubled in the last decade, and US emergency department visits related to food allergy have also significantly increased ^3^. Death caused by food-induced anaphylaxis is extremely rare ^4^, but peanuts and tree nuts are believed to cause a disproportionate share of these deaths, more than 90% by some estimates ^4^, and as many as 18% to 40% of all food-induced anaphylactic reactions are estimated to be caused by tree nuts ^5, 6^. The prevalence of both tree nut and peanut allergy appears to be increasing, at least in the pediatric population; a survey of 5,300 households found that the prevalence of tree nut or peanut allergy rose from 1.2% in 2002 to 2.1% in 2008 ^7^. Walnuts, hazelnuts, cashews, and almonds are the tree nuts that most likely cause allergic reactions. For both peanuts and many tree nuts, the quantity required to trigger an allergic reaction (50% of the maximum response, or ED _50_) is very low compared with other major food allergens ^8^.

Given the ubiquity of nuts in Western diets and the rising prevalence of nut allergy, it is unsurprising that stories about fatal nut-related anaphylaxis have gained traction in the lay press. News articles, such as those written about a Canadian woman who died after a peanut-laced kiss, further heighten public anxiety about nut allergy ^9^. Many elementary schools in the US have responded to increased concerns about nuts by instituting “nut-free zones” and policies. Blogs and websites like “No Nuts Moms Group” promoting nut-free policies have proliferated. Nut allergy is associated with significant psychological burden ^10^ and has a negative impact on quality of life ^11^. Patients with nut allergy are subject to increased stress and anxiety, as ordinary activities such as grocery shopping or eating at a restaurant can provoke fear ^10^.

## Allergic potential of peanuts and tree nuts

Peanuts ( *Arachis hypogea*) and tree nuts are seeds and, as such, contain numerous energy storage proteins. Botanically, neither peanuts nor many tree nuts are true nuts. Peanuts are legumes, a group that also includes peas, beans, chickpeas, soybeans, alfalfa, and clover. Hazelnuts and chestnuts are true nuts, composed of an edible seed surrounded by a hard shell, which is surrounded by a woody protective layer. Most other tree nuts are drupe seeds. Drupes, also known as stone fruits, comprise a category of botanical fruit that includes the culinary fruits apricots, peaches, mangos, and nectarines. The seeds of these fruits are housed in a hardened exocarp or “pit”, which in turn is typically surrounded by juicy flesh and thin skin. Pecans, walnuts, almonds, cashews, and pistachios are all considered drupe seeds rather than nuts.

At least three broad categories of seed storage protein have been identified as potentially being important in food allergy: 2S albumins, vicilins (7S globulins), and legumins (11S globulins). In peanuts, Ara h 1 is a vicilin, Ara h 2 is a 2S albumin, and Ara h 3 is a legumin; of these, Ara h 2 is likely responsible for most anaphylactic reactions ^12, 13^. Patients with peanut allergy may have a positive skin prick test (SPT) or serum test to tree nut extract as the result of cross-sensitization, in which epitope similarity allows a single immunoglobulin to bind to different proteins ^14^.

One factor contributing to the allergenicity of peanuts and tree nuts is their ability to activate the innate immune system. For instance, group 2 innate lymphoid cells are increased in peanut-allergic patients compared with non-atopic controls ^15^. Furthermore, peanuts have been shown to contribute to shock in mice by stimulating the production of the complement component C3a, independently of any adaptive immune response to peanut proteins ^16^. Innate immune cells, including dendritic cells, macrophages, and natural killer cells, express pattern recognition receptors (PRRs), including Toll-like receptors, Nod-like receptors, and Rig-like receptors, which often bind to substances that are present in injury or infection. Substances that enhance the innate immune response to an antigen, particularly through the stimulation of PRRs, are referred to as adjuvants. Examples of well-characterized adjuvants are lipopolysaccharide (LPS), which is produced by Gram-negative bacteria, and beta glucan, which is produced by fungi. There is some evidence that highly allergenic drupe seeds and legumes may promote innate immune activation in a way that less-allergenic legumes do not. One experiment showed that peanut extract injected into a mouse foot led to increased cell number and cytokine production in the ipsilateral popliteal lymph node ^17^. In another study, peanut exposure to abraded mouse skin also increased innate cytokine peanut-specific IgE/IgG1 production, even without the addition of an exogenous adjuvant ^18^. In the same study, cashew exposure without adjuvant also led to the generation of cashew-specific IgE/IgG1 ^18^. However, mice exposed to an extract of green bean (a less-allergenic legume) did not develop a similar IgE response unless cholera toxin, an established exogenous adjuvant, was added to the solution ^18^.

## Use of component-resolved diagnostics for peanut and tree nut allergies

Specific IgE immunoassays (sIgE) (described above) may yield a positive result even when a patient does not have a clinically significant allergy to the allergen in question. The sIgE technique uses peanut or tree nut extract. However, not all peanut and tree nut proteins are equally allergenic, and the presence of IgE to some proteins is more strongly associated with severe allergy than others. With peanuts, patients with sIgE to the “anaphylactogenic” protein Ara h 2 are more likely to develop severe, systemic reactions than patients with Ara h 8, as Ara h 8 is homologous to Bet v 1 and other birch pollen allergens and therefore associated with the milder oral allergy syndrome ^19^. Component-resolved diagnostics (CRD) use recombinant protein immunoassays rather than extracts to determine which specific proteins patient antibodies are reactive to. In a patient with a positive sIgE to peanut, CRD can elucidate which of the Ara proteins the patient’s antibodies are reactive to and therefore can predict the clinical severity of the patient’s allergy. Early studies indicate that CRD is a promising tool for making such predictions ^20^. A recent experiment involving the use of CRD in 108 patients with peanut allergy suggested that less than half of peanut-allergic patients who avoid tree nuts are also likely to be tree nut allergic on the basis of the results of CRD testing for tree nuts. However, this study did not perform oral food challenge (OFC) with the tree nuts tested, so the clinical significance of these findings is yet to be determined ^21^.

## Risk factors for peanut and tree nut allergy development

Numerous risk factors for the development of peanut and tree nut allergy have been identified, including family history of peanut allergy ^22, 23^, personal history of egg allergy, and personal history of atopic dermatitis ^24^, including filaggrin mutations ^25^. More controversial associations include maternal ingestion of peanut during pregnancy and lactation ^26, 27^ and soy consumption in infancy ^28, 29^. Family history of peanut allergy is a long-established risk factor for peanut allergy ^24^; however, many studies identifying family history as a risk factor took place in the era before early introduction was widely recommended ^30^. As such, delayed oral exposure to peanut may have contributed to increased peanut allergy risk in children whose family members had peanut allergy in some studies.

Whereas early oral exposure appears to protect against peanut allergy development, multiple lines of indirect evidence suggest that early cutaneous exposure to peanuts is sensitizing. Mice exposed to peanut extract through the cutaneous route were found to become sensitized in a T helper type 2 (Th2)-dependent manner ^18^. In one questionnaire-based study investigating 133 peanut allergy cases and 150 non-allergic controls, median weekly household peanut consumption (MWHP) was 18.8 g in the peanut-allergic group compared with 6.9 g in the non-allergic controls ^31^, suggesting that children in households in which more peanut products were consumed were at higher risk of developing peanut allergy, perhaps through cutaneous contact with peanut particles.

Though less exhaustively studied than those for peanut allergy, the risk factors for tree nut allergy are similar to those for peanut allergy. In one registry of 5,149 mostly pediatric patients with legume and tree nut allergy, 1,667 were found to be tree nut allergic ^32^. Of these, walnut allergy was most common (34%), followed by cashew (20%), almond (15%), pecan (9%), and pistachio (7%) ^32^. Allergies to hazelnut, pine nut, and macadamia occurred among less than 5% of the tree nut-allergic group.

## Natural history of peanut and tree nut allergy in childhood

Experts have long considered peanut and tree nut allergy to be highly persistent. Owing in part to the severity of peanut allergy, providers have been hesitant to re-introduce peanuts to patients with a history of peanut allergy. A 1989 study involving 32 subjects with food challenge-proven peanut allergy measured skin reactivity as its primary means of determining the persistence of peanut allergy and concluded that none of the study subjects had outgrown their allergy. However, newer data contradict this paradigm, indicating that as many as 21.5% of children with peanut allergy are able to tolerate peanuts at an increased age ^33^. A subsequent study showed that in the subgroup of peanut-allergic children with initial peanut sIgE of not more than 5 kUA/L, more than half can be expected to outgrow their allergy ^34^. Generally, higher SPT and peanut-specific sIgE appear to correlate with more-persistent allergy whereas lower values indicate allergy that is more likely to fade with time. One study found that SPT 6 mm or greater and peanut sIgE 3 kUA/L were each predictive of persistent peanut allergy ^35^. Another found that low peanut sIgE and decreasing SPT size between the ages of 1 and 4 were crucial determinants that foretold remission or resolution of peanut allergy ^36^. This study also found that when peanut allergy did resolve, it was most likely to occur before the age of 8 ^36^.

There are fewer data on the natural history of tree nut allergy. One study involving 278 patients with tree nut allergy found that only 9% of the overall group outgrew their allergy. However, as with peanut allergies, sIgE appeared to correlate well with prognosis, as 63% of patients with sIgE to tree nuts less than 2 kUA/L and 75% of patients with negative sIgE outgrew their allergy ^37^.

## Understanding the pathophysiology of nut allergy

Given the recent increase in the prevalence of nut allergy, there has been great interest in understanding the pathogenesis of this disease state. Numerous explanations have been offered for the global rise in nut allergy, but no single hypothesis offers a unifying theory of disease.

The hygiene hypothesis, which has its basis in the observation that Th2-mediated diseases like asthma and eczema are less common in developing countries, has been offered as an explanation for increased food allergy in developed countries ^38^. In the absence of frequent infection, and exposure of the immune system to a variety of microbial antigens, there is dysregulation of Th1, Th2, and regulatory T (Treg) responses. The hygiene hypothesis, however, cannot fully account for the increasing burden of food allergy ^39^.

Emerging research on the cutaneous and enteric microbiome has led some researchers to argue that the term “hygiene hypothesis” is too narrow and that immune development is influenced not only by helminths but also by bacterial and viral organisms. Some prefer the term “biome depletion” for its more inclusive scope ^40^. Specific commensal bacterial species (that is, *Bacteroides* and *Lactobacillus*) have been associated with decreased risk of atopic disease in animal models ^41^, and the chemical metabolites produced by these organisms (particularly short-chain fatty acids) have been associated with beneficial effects on allergy and atopy ^42^.

Furthermore, the gut microbiome may play an important role in the development of peanut and tree nut allergy. In one study, mice given antibiotics early in life were more likely to become sensitized to peanuts, and further experiments suggested that *Clostridium* species in particular were able to “shield” highly allergenic peanut proteins from the bloodstream ^43^. These findings have led to the hypothesis that increased antibiotic exposure may in part explain the recent rise in peanut allergy in children ^42^.

Other hypotheses about the development of allergy have been cultivated on the basis of animal research and epidemiologic studies. There is some evidence to suggest a benefit associated with omega-3 and omega-6 fatty acid supplementation, although a recent Cochrane review showed no clear benefit of fatty acid supplementation during childhood in human studies ^44^. Antioxidants may also protect against allergy development ^45^.

Skin exposure to food allergens in early childhood is another intriguing hypothesis for the development of allergy. This hypothesis stems from the observation that antigens introduced to the skin of experimental animals are more likely to provoke an allergic response than antigens exposed to the oral mucosa or gastrointestinal tract. Peanut antigens, in particular, seem to be particularly effective at eliciting an allergic immune reaction in murine skin. Mice exposed to peanut extract or Ara h 2 on skin frequently develop a Th2 response ^18, 46^.

More research is needed to elucidate the pathogenesis of nut allergies and of food allergies generally. Overall, a Th2 response and immune dysregulation play an important role, a finding that has led to the “biome depletion” and the “hygiene hypothesis”.

## The avoidance paradigm: journey to the American Academy of Pediatrics guidelines

In the 1990s, amidst a growing realization that food allergy was becoming more common worldwide, one study found that parents of patients with clinical peanut allergy were more likely to report the consumption of peanuts during pregnancy and early introduction of peanut products ^47^. Despite the potential for recall bias in such lines of inquiry (that is, mothers whose children exhibited clinical peanut allergy may have been more likely to recall the consumption of peanuts), these findings were consistent with other results being published at the time. A large study from New Zealand found that early introduction of solid foods appeared to be correlated with eczema, which in turn was known to be associated with food allergy ^48^. These and other lines of indirect evidence were considered in drafting the 1999 European Society for Pediatric Allergology and Clinical Immunology (ESPACI) and the European Society for Pediatric Gastroenterology, Hepatology, and Nutrition (ESPGHAN) guidelines for the prevention of food allergy. In 2000, the American Academy of Pediatrics (AAP) introduced parallel guidelines for the same purpose. High-risk infants were defined as those with a parent or sibling with food allergy. The 2000 AAP guidelines recommended that mothers breastfeeding high-risk infants eliminate peanuts and tree nuts from their diet and that peanuts and tree nuts be avoided until 36 months of age. Notably, the latter recommendation was based on expert consensus rather than any clinical evidence; it was a significant departure from the ESPACI/ESPGHAN guidelines, which were far less restrictive and only recommended the avoidance of peanuts and tree nuts until 5 months of age. In its reasoning, the AAP reflected the contemporary thinking: that because peanut avoidance alone was unlikely to lead to nutritional deficiency, the potential benefit of peanut avoidance was likely to outweigh any disadvantages.

## Paradigm shift: the LEAP, LEAP-On, and EAT studies

One crucial clue regarding the etiology of nut allergy came from the observation by British researchers in the mid-2000s that Ashkenazi Jewish children in the UK had far higher rates of peanut allergy than did Ashkenazi Jewish children in Israel ^49^. Inquiry led to the insight that the UK children typically avoided peanut products in childhood but that the Israeli children were often given Bamba, a confection made from popped corn grits soaked in peanut butter, as an early solid food. In the Learning Early About Peanut Allergy (LEAP) study, Du Toit *et al*. ^50^ selected infants at high risk for peanut allergy for inclusion in a single-site, prospective randomized design. During the initial screening, infants who had severe eczema or egg allergy were determined to be at high risk for developing peanut allergy and were selected for the trial ^50, 51^. SPT was performed, and 76 individuals with wheal size of more than 4 mm were excluded from the trial ^50^. Of the remaining 640 participants, 530 were found to have a 0 mm wheal on SPT and 98 had 1 to 4 mm wheals on SPT. Subjects were randomly assigned to peanut avoidance until age 5 or consumption of 6 g of peanut protein from enrollment until age 5. Even when baseline SPT results were accounted for, subjects randomly assigned to peanut consumption exhibited dramatically lower rates of clinical peanut allergy by age 5 (2% versus 14% in the 0 mm wheal group and 10% versus 35% in the 1 to 4 mm group) ( Table 1). In the LEAP-On study, participants from the LEAP study were instructed to avoid peanut consumption for 12 months. Twelve months of peanut avoidance did not significantly increase the rate of clinical peanut allergy in subjects who had been in the peanut consumption group during the LEAP study. At the conclusion of the LEAP-On study, the children who had been assigned to peanut consumption in the LEAP study had a 4.8% peanut allergy rate compared with an 18.6% peanut allergy rate in children who had been assigned to peanut avoidance. The findings of the LEAP-On study indicate that the benefit conferred by peanut product consumption in early life is lasting and durable, even if peanut consumption is paused later in childhood.

**Table 1.** Percentage of infants who developed clinical peanut allergy in a 5-year study window, stratified by wheal size ascertained before intervention.

0 mm wheal (n = 530)	Consumption	1.9%
	Avoidance	13.7%
1–4 mm wheal (n = 98)	Consumption	10.3%
	Avoidance	35.3%

Data are from the Learning Early About Peanut Allergy (LEAP) study. Adapted from Du Toit *et al*. ^51^.

The LEAP and LEAP-On studies involved only high-risk infants and peanuts. The Enquiring About Tolerance (EAT) study ^52^, involving 1,303 infants in the UK, was a randomized prospective study designed to determine whether the early introduction of peanut, cooked egg, cow’s milk, sesame, whitefish, and wheat could reduce the occurrence of food allergy regardless of *a priori* allergy risk. This study, like LEAP, found a significant reduction in the incidence of peanut allergy with early introduction (2.4% with early introduction versus 7.3% with standard introduction) ^52^.

## Implications for clinical practice: 2017 National Institute of Allergy and Infectious Diseases guidelines

**Figure 1.**
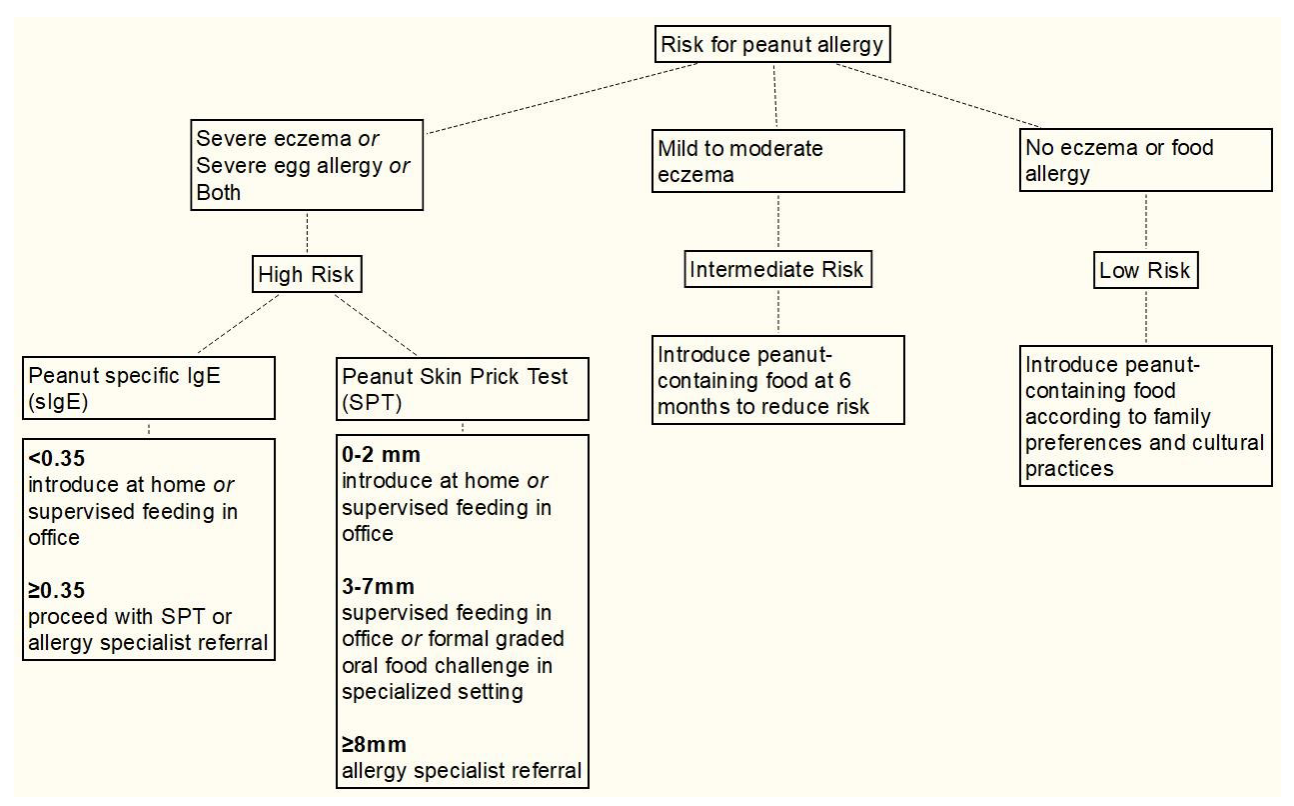
National Institute of Allergy and Infectious Diseases algorithm for risk stratification, evaluation, and management of infants with respect to peanut allergy. Adapted from Togias *et al*. ^53^.

In 2017, the National Institute of Allergy and Infectious Diseases (NIAID) released guidelines for the early introduction of peanut in selected children. These guidelines were heavily influenced by the findings of the LEAP study. The NIAID guidelines (summarized in Figure 1) constitute an algorithmic approach to early peanut introduction. The first step in this algorithm is risk-stratifying the patient. Infants with severe eczema or severe egg allergy or both are at highest risk of developing peanut allergy, and those in this “high-risk” group should undergo testing as soon as possible to determine whether they are already sensitized.

For “high-risk” infants, most practitioners should begin by ordering a peanut-specific IgE (sIgE). If this is less than 0.35, which is most common, the risk of existing allergy is low and peanut products can be introduced at home or—for parents who are more concerned—in the office. For all “high-risk” infants, peanut introduction should occur as soon as possible, ideally at age 4 to 6 months. If the sIgE is at least 0.35, then SPT or allergy specialist referral is indicated. If SPT shows 0 to 2 mm induration, then home introduction of peanut product is suitable. If SPT shows 3 to 7 mm induration, then supervised feeding in the office or a formal OFC is appropriate. Allergy specialist referral is warranted for infants who exhibit at least 8 mm induration on SPT because these infants are likely to have clinical peanut allergy. Peanuts should not be given to infants with at least 8 mm SPT induration prior to specialist evaluation and management. Providers should keep in mind that, for high-risk infants, the process of evaluation should begin as early as possible, as it can take some time for a SPT to be performed and peanuts should be introduced as early as 4 months in this group.

Most providers should begin with sIgE and consider SPT if sIgE is consistent with peanut sensitization. SPT is considered more precise by some. However, neither SPT nor sIgE alone can provide a definitive diagnosis; this requires clinical history that is consistent with food allergy or OFC. Infants with mild to moderate eczema but no severe egg allergy are considered to be at “intermediate risk” for peanut allergy. Unlike infants in the “high-risk” group, these infants do not need to undergo sIgE testing or SPT. They should be given peanut-containing foods at 6 months. No special evaluation or treatment is needed for infants who do not have eczema or food allergy. These infants constitute the “low-risk” group.

In the LEAP study, Bamba (described earlier) was used and is a safe and suitable option for most infants, but peanut powders that can be mixed into applesauce or porridge are now commercially available. A prepackaged mixture of applesauce and peanut (My Peanut™) is also commercially available. Smooth peanut butter diluted with water or applesauce has also been used in some instances. Non-diluted peanut butter, whole peanuts, and crushed peanuts represent choking hazards and are *not* suitable for the early introduction of peanut.

## Conclusions and future directions

Although our understanding of peanut allergy pathogenesis remains incomplete, the LEAP study and NIAID guidelines offer a clear departure from long-established practices. There are numerous challenges to the real-world implementation of these guidelines, most notably the problem of SPT or peanut-specific serum IgE testing, whose specificity and sensitivity in predicting clinical food allergy are suboptimal. Furthermore, access to allergy specialists, who are typically in great demand, may be a limiting factor for “high-risk” infants who require SPT prior to the initiation of peanut consumption at 4 to 6 months. The NIAID guidelines do not explicitly recommend a dose for early peanut consumption, but the original LEAP study used 6 g of peanut protein per week, which translated to 21 Bamba sticks per week, split into three feedings of seven sticks. Another challenge is the issue of siblings with peanut allergies; siblings of a child with peanut allergy are at sevenfold increased risk of developing peanut allergy ^54^. If an infant’s older sibling has peanut allergy, then regular feedings of peanut powder or Bamba sticks could be a source of anxiety for parents and caregivers. Such concerns are valid, and more public health and health systems research is needed to determine better ways to offer families timely, safe, and affordable access to evaluation and management for peanut allergy prevention. In the meantime, providers should take steps to offer early introduction of peanut where it is appropriate.

## Abbreviations

AAP, American Academy of Pediatrics; CRD, component-resolved diagnostics; ESPACI, European Society for Pediatric Allergology and Clinical Immunology; ESPGHAN, European Society for Pediatric Gastroenterology, Hepatology, and Nutrition; LEAP, Learning Early About Peanut Allergy; NIAID, National Institute of Allergy and Infectious Diseases; OFC, oral food challenge; PRR, pattern recognition receptor; sIgE, specific IgE; SPT, skin prick test; Th, T helper
